# MetaChest: generalized few-shot learning of pathologies from chest X-rays

**DOI:** 10.1186/s42492-026-00214-4

**Published:** 2026-02-06

**Authors:** Berenice Montalvo-Lezama, Gibran Fuentes-Pineda

**Affiliations:** 1 Posgrado en Ciencia e Ingeniería de la Computación, Universidad Nacional Autónoma de México, Circuito Escolar s/n, Ciudad Universitaria, Coyoacán, 04510 CDMX México; 2 Instituto de Investigaciones en Matemáticas Aplicadas y en Sistemas, Universidad Nacional Autónoma de México, Circuito Escolar s/n, Ciudad Universitaria, Coyoacán, 04510 CDMX México

**Keywords:** Few-shot learning, Chest X-ray dataset, Chest X-ray multi-label classification, Meta-learning, Deep learning

## Abstract

The limited availability of annotated data presents a major challenge in applying deep learning methods to medical image analysis. Few-shot learning methods aim to recognize new classes from only a few labeled examples. These methods are typically investigated within a standard few-shot learning paradigm, in which all classes in a task are new. However, medical applications, such as pathology classification from chest X-rays, often require learning new classes while simultaneously leveraging the knowledge of previously known ones, a scenario more closely aligned with generalized few-shot classification. Despite its practical relevance, few-shot learning has rarely been investigated in this context. This study presents MetaChest, a large-scale dataset of 479,215 chest X-rays collected from four public databases. It includes a meta-set partition specifically designed for standard few-shot classification, as well as an algorithm for generating multi-label episodes. Extensive experiments were conducted to evaluate both the standard transfer learning (TL) approach and an extension of ProtoNet across a wide range of few-shot multi-label classification tasks. The results indicate that increasing the number of classes per episode and the number of training examples per class improves the classification performance. Notably, the TL approach consistently outperformed the ProtoNet extension, even though it was not specifically tailored for few-shot learning. Furthermore, higher-resolution images improved the accuracy at the cost of additional computation, whereas efficient model architectures achieved performances comparable to larger models with significantly reduced resource requirements.

## Introduction

In recent decades, deep learning has revolutionized medical image analysis, particularly in radiology [1–3]. Deep neural networks have enabled the processing of large volumes of radiological data, the extraction of complex features, and the development of models that can enhance the accuracy of medical diagnoses. Despite these advances, a major challenge arises when only limited annotated data are available because deep learning models typically require large amounts of labeled data to achieve strong performance. This issue is particularly relevant to tasks, such as pathology classification in chest X-rays, where labeled data can be scarce and difficult to obtain. To address this limitation, early research explored the standard few-shot classification (SFSC) paradigm, which aims to train models capable of generalizing to new classes using only a few labeled examples per class. However, this paradigm significantly differs from the manner in which pathologies manifest in practice. From a clinical perspective, the objective is not merely to classify entirely new disease categories but rather to distinguish between a combination of known and previously unseen pathologies. This highlights the need for approaches more closely aligned with the complexities of clinical settings.

This study investigates the factors influencing the training of pathology classification models using a formulation that more closely reflects clinical scenarios. In particular, how different task instance configurations within the generalized few-shot learning (GFSL) paradigm affect model performance is studied. In addition, two training methods derived from standard transfer and standard few-shot learning (SFSL) paradigms are compared and evaluated on tasks with a GFSL formulation. Finally, the impact of image resolution and neural network architecture on classification performance is analyzed.

To address these objectives, this work makes the following contributions.

- MetaChest, a dataset comprising 479,215 chest X-ray images collected from four public databases, is introduced, along with a meta-set partition specifically designed for SFSC.
- An algorithm to generate multi-label episodes is provided, enabling few-shot learning in multi-label settings.
- ProtoNet-ML, an extension of ProtoNet for multi-label classification tasks, is proposed.
- A comprehensive comparison of two methods, one based on standard transfer learning (STL) and the other on SFSL, is conducted across a wide range of tasks with varying complexity.
- The influence of image resolution and model architecture on pathology classification performance is analyzed.

The remainder of this paper is organized as follows: Methods section introduces the MetaChest dataset and the key differences between STL, SFSC, and generalized few-shot classification (GFSC) are outlined. A multi-label episode generation algorithm is introduced and two classification methods used in this work: BatchBased and ProtoNet-ML are described. Results and Discussion section presents and analyzes the experimental results. Finally, Conclusions section summarizes the conclusions and outlines the directions for future research.

### Related work

The related studies on chest X-ray classification using deep learning techniques are reviewed. Relevant transfer learning (TL) and meta-learning (MTL) approaches are also discussed, as well as their applications to the medical image domain.

#### Deep learning for chest X-ray classification

Deep neural networks, coupled with large-scale datasets, have enabled significant progress in several computer vision fields. Over the past few years, datasets of increasingly specialized domains have become publicly available for deep neural networks. For instance, in the medical domain, multiple chest X-ray datasets have been introduced, such as CheXpert [4], Chest X-ray8 [5], Chest X-ray14 [5], MIMIC [6], MIMIC-CXR-JPG [7], OpenI [8], and PadChest [9]. These datasets have been fundamental to the development of deep learning models for chest X-ray analysis and generation tasks. In contrast to ImageNet [10], the scales of these datasets are at least one order of magnitude smaller. Additionally, the distributions of these datasets are highly heterogeneous. This includes the number and type of pathologies, class imbalance, collection and labeling procedures, image quality, and patient population.

With the introduction of these datasets, several studies addressing pathology classification from chest X-rays using deep learning have been conducted. For binary classification (i.e., presence or absence), Lakhani and Sundaram [11] focused on tuberculosis identification, whereas Mabrouk et al. [12] focused on pneumonia identification. Because the X-rays of a patient may exhibit signs of multiple diseases, the identification of pathologies from chest X-rays has often been formulated as a multi-label classification problem. For instance, Baltruschat et al. [13] used the ResNet-50 [14] architecture to classify 14 pathologies in the ChestX-ray14 dataset, where each X-ray can be assigned to more than one pathology. Similarly, Irvin et al. [4] compared various ConvNet architectures for multi-label chest X-ray classification using CheXpert and found that DenseNet121 outperformed ResNet152 [14], Inception-v4 [15], and SE-ResNeXt101 [16].

#### STL

TL is the cornerstone of deep learning for image analysis because it can reduce the amount of data and computational resources required to train a model for a target task by leveraging representations learned from one or multiple source tasks. In TL, multiple strategies have been proposed for adapting representations from a source task to a target task. In practice, the most widely used transfer strategy has been STL, which consists of pre-training models that use conventional batch-based training (as opposed to other training schemes). Specifically, ImageNet pre-training has been a standard practice for a wide variety of natural image tasks, including classification [17, 18], segmentation [19, 20], and object detection [21].

Owing to the widespread use of ImageNet in practice, multiple studies have examined the transferability of learned representations from ImageNet to other natural image tasks [22–25]. Surprisingly, although some studies have suggested that the source and target datasets must be closely related for effective knowledge transfer [23], ImageNet pre-training has been successfully used for widely dissimilar image domains (e.g., medical images [2, 5, 26]). By contrast, transferability studies in specific domains exist, in which ImageNet pre-training does not provide any improvement over random initialization [1, 3, 26].

Other transferability studies have focused on analyzing the effects of the architecture size and scale of the training dataset on the effectiveness of STL. In intra-domain scenarios where the source and target datasets are closely related, studies have mainly focused on natural image datasets [3, 18, 27]. For example, Kolesnikov et al. [18] and Zhai et al. [27] analyzed how the pre-training dataset size and architecture depth influence knowledge transfer when both the source and target datasets are composed of natural images. The results of these studies consistently demonstrated better performance with larger architectures and pre-training datasets.

In inter-domain scenarios, where the source and target datasets belong to different domains (e.g., natural images and chest X-rays), existing studies are scarce, not very systematic, and report mixed results. Raghu et al. [26] did not find significant differences on chest X-ray and retinal image classification performance using a ResNet-50 architecture when comparing ImageNet-1K pre-training with random initialization. Ke et al. [2] studied the effect of ImageNet-1K pre-training on chest X-ray classification performance using ConvNet architectures of different sizes. Their results showed a slight performance improvement when deeper pre-trained architectures were used. By contrast, Mustafa et al. [1] studied the influence of ImageNet-1K, ImageNet-21K, and JFT-300M pre-training on the classification performance using ResNets of different sizes. The target tasks considered in this study were cancer identification from mammograms, pathology classification from chest X-rays, and skin condition identification from dermatological images. The results were inconclusive, exhibiting performance improvements with larger pre-training datasets and architectures only for certain target tasks. Similarly, Cherti and Jitsev [3] conducted a comparative study of ResNet models pre-trained on ImageNet-1K, ImageNet-21K, and a combination of different chest X-ray datasets for pathology classification. They reported small performance improvements when the models were pre-trained on larger source datasets and transferred to larger target datasets. However, no performance improvement was observed when transferring smaller target datasets, regardless of the size of the pre-training dataset.

#### Few-shot classification

MTL is a TL strategy for generating models that can quickly adapt to new tasks [28]. In contrast to STL, in MTL, new tasks, commonly known as episodes, are typically small with respect to both the number of classes and examples per class. The most widely studied problem in MTL is few-shot classification, which is a multi-class classification problem in which only a few examples per class are available for training (typically 1 or 5).

The earliest works on MTL proposed methods for multi-class classification of natural image datasets [29–33], such as MiniImageNet [30] and FC100 [34]. These datasets are reduced versions of ImageNet and CIFAR100 and were created to facilitate episodic training.

More recent studies have applied MTL to domain-specific problems, particularly using datasets from various medical imaging modalities. For example, MTL methods have been studied for skin disease classification using dermatological images [35], COVID-19 classification using chest computed tomography (CT) scans [36], and cancer classification using histological images [37, 38]. Moreover, MTL methods have been used for image segmentation in CT scans, magnetic resonance images [39], and dermatological images [40].

## Methods

This section introduces the MetaChest dataset, the TL strategies adopted, the task formulation, and the classification methods used.

### MetaChest dataset

Over the past few decades, several chest X-ray datasets have been collected, which vary in terms of the number of examples, study population, labeling strategy, period of time, pathologies, and source institution. Table 1 shows a comparison of publicly available chest X-ray datasets. In general, these datasets exhibit heterogeneous characteristics, ranging from a few thousand to hundreds of thousands of images collected over periods of a few years to a few decades. One key factor influencing the distribution of pathologies in a dataset is the patient population from which chest X-rays were obtained. As observed in Table 1, most publicly available datasets were collected from medical institutions in the United States, albeit from different hospitals and regions. However, there are two datasets from other countries: PadChest from Hospital San Juan in Spain and VinDr-CXR from multiple hospitals in Vietnam.

**Table 1:** Comparison of publicly available chest X-ray datasets

Dataset	Number of pathology	Number of image	Period	Source	Labeling pipeline
OpenI	18	7,470	NA	Indiana Network for Patient Care, USA	MeSH
ChestX-ray8	8	108,948	1992–2015	National Institutes of Health, USA	MetaMap, DNorm, custom negation rules
ChestX-ray14	14	112,120	1992–2015	National Institutes of Health, USA	MetaMap, DNorm, custom negation rules
CheXpert	14	224,316	2002–2017	Stanford Hospital, California, USA	CheXpert
MIMIC	14	377,110	2011–2016	Beth Israel Deaconess Medical Center, USA	CheXpert/NegBio
PadChest	19	168,861	2009–2017	Hospital San Juan Alicante, Spain	Physicians
VinDr-CXR	14	18,000	2018–2020	Hanoi Medical University Hospital and Hospital 108, Vietnam	VinDr Lab

MIMIC [6] refers to MIMIC-CXR-JPG [7]

Clinical data collection is a complex process that involves several tasks requiring considerable time and resources. Data labeling is one of the tasks that can be a source of greater variability among chest X-ray datasets. The rightmost column in Table 1 summarizes the labeling strategy employed by each dataset. Most datasets automatically derived annotations from radiology reports using natural language processing methods, except for PadChest, which was annotated by expert radiologists. The specific strategy and tool used for annotating the chest X-rays directly influence the distribution of labels. For instance, MIMIC provides two sets of labels with different distributions: one generated by NegBio [41] and the other by CheXpert [4].

An inherent characteristic of medical datasets is class imbalance; that is, the number of examples associated with one pathology is significantly larger than that associated with other pathologies. This is due to multiple factors, including the prevalence of each pathology in the study population or even the severity of the pathology (which could lead to multiple subsequent chest X-rays).

#### Data

To obtain a dataset with a more general epidemiological distribution for evaluating pathology classification models trained on a few examples, MetaChest, a combination of CheXpert, MIMIC, ChestX-ray14, and PadChest, is proposed. It provides a MTL oriented partitioning suitable for few-shot learning scenarios. Only patients aged between 10 and 80 years were considered, and incomplete records and corrupted images were discarded. Overall, MetaChest comprises 479,215 chest X-ray images, of which 322,475 are multi-labeled. Each image is associated with one or more of the 15 most common pathologies across the four original datasets, resulting in 596,494 different pathology instances. By contrast, 156,740 images are normal or labeled as not finding, indicating that no specific abnormalities were observed in the original datasets.

**Fig. 1:**
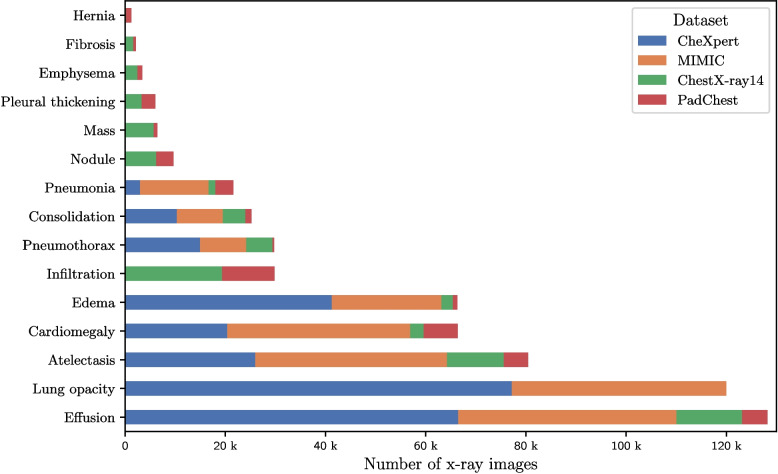
Distribution of labels per pathology and dataset in MetaChest

The frequencies of each pathology in MetaChest are shown in Fig. 1. As observed, there is a pronounced class imbalance, with the most frequent pathology (Effusion) occurring nearly two orders of magnitude more often than the least frequent (Hernia). With respect to labeling, MetaChest has a label cardinality (average number of labels per image) of 1.84 and a label density (average number of labels per image over total number of classes; see Tsoumakas et al. [42]) of 0.12.

**Fig. 2:**
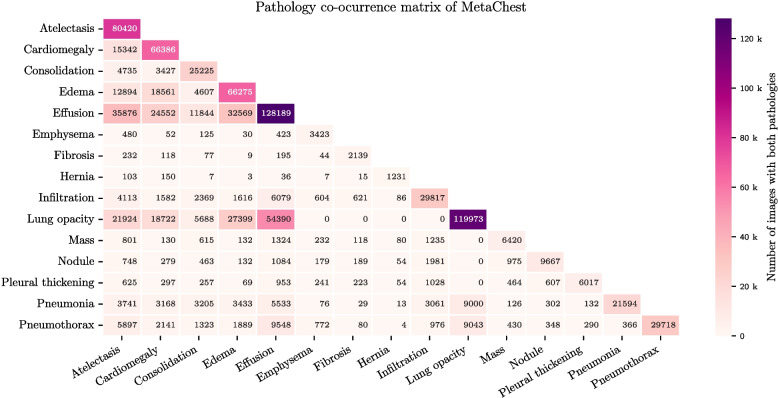
Co-occurrence matrix of MetaChest pathologies

The co-occurrence of labels in MetaChest is illustrated in Fig. 2. The most frequently co-occurring pathology pairs are Lung Opacity-Effusion, Effusion-Atelectasis, and Effusion-Edema. Although Lung Opacity is the second most frequent pathology in MetaChest and frequently occurs together with five pathologies, seven pathologies never present together. Moreover, Hernia is a pathology that occurs less commonly together with other pathologies, which is expected because it is also the less frequent pathology in MetaChest.

The code used to generate MetaChest is publicly available at https://github.com/bereml/metachest and on the dataset’s website at https://bereml.github.io/metachest/.

#### MTL partition

This study focuses on pathology classification using small datasets with a few classes and a few examples per class. In particular, an episode-based setting similar to SFSL was considered, where the classifier is trained and evaluated across multiple episodes to study the model’s behavior in scenarios with a small number of classes and few examples.

Therefore, MetaChest classes were partitioned into meta-training $\mathcal {C}_{meta-trn}$, meta-validation $\mathcal {C}_{meta-val}$, and meta-test $\mathcal {C}_{meta-tst}$ sets using the following procedure. First, for $\mathcal {C}_{meta-tst}$, the five pathologies with the fewest images present in all four original datasets were selected. This allows the study of the dataset shift and its impact on classification performance. Subsequently, from the remaining nine pathologies, the five with the largest number of images were selected for $\mathcal {C}_{meta-trn}$ and the other four for $\mathcal {C}_{meta-val}$. Unlike the meta-test set, the meta-training and meta-validation sets comprised pathologies that were not available in all of the four original datasets.

Table 2 shows the classes associated with each meta-set, along with the number of examples contributed by each original dataset. In general, CheXpert and MIMIC provide the largest number of labels for the meta-training and meta-test sets; together, these two datasets account for 77.33% and 86.24% of the total labels in the meta-training and meta-test sets, respectively. By contrast, ChestX-ray14 and PadChest provide all the examples in the meta-validation set, and of several pathologies in the meta-training set. This is because of the absence of these pathologies in CheXpert and MIMIC. Although CheXpert contributes to only three different pathologies (Effusion, Lung Opacity, and Atelectasis) in the meta-training set, it accounts for 44% of the total labels in this meta-set. By contrast, both PadChest and ChestX-ray14 contribute to six out of seven pathologies yet cover only 7.14% and 15.51% of the total labels in the meta-training set, respectively. There are 99,983 normal images in the meta-training set, 1,788 in the meta-validation set, and 54,969 in the meta-test set.

**Table 2:** Meta-training, meta-validation, and meta-test class sets with the corresponding number of label instances per pathology

Class	MetaChest	Dataset			
		CheXpert	MIMIC	ChestX-ray14	PadChest
$$\mathcal {C}_{meta-trn}$$					
Effusion	128,189	66,484	43,544	13,086	5,075
Lung opacity	119,973	77,194	42,779		
Atelectasis	80,420	25,980	38,297	11,335	4,808
Infiltration	29,817			19,362	10,455
Nodule	9,667			6,238	3,429
Mass	6,420			5,682	738
Pleural thickening	6,017			3,326	2,691
*Total*	*380,503*	*169,658*	*124,620*	*59,029*	*27,196*
$$\mathcal {C}_{meta-val}$$					
Emphysema	3,423			2,484	939
Fibrosis	2,139			1,650	489
Hernia	1,231			197	1,034
*Total*	*6,793*			*4,331*	*2,462*
$$\mathcal {C}_{meta-tst}$$					
Cardiomegaly	66,386	20,391	36,512	2,701	6,782
Edema	66,275	41,247	21,894	2,269	865
Pneumothorax	29,718	14,977	9,215	5,220	306
Consolidation	25,225	10,340	9,183	4,505	1,197
Pneumonia	21,594	2,986	13,679	1,381	3,548
*Total*	*209,198*	*89,941*	*90,483*	*16,076*	*12,698*

Although Cherti and Jitsev [3] used a chest X-ray dataset that combined multiple datasets, it is not publicly available, its generation procedure is not described, and appropriate partitions for the MTL evaluation are not provided. Similarly, TorchXRayVision [43] is a library that allows the combination of different chest X-ray datasets but does not consider SFSL scenarios. Conversely, MetaChest employs a disjoint class partition that enables experimentation in SFSL settings. In addition, $\mathcal {C}_{meta-tst}$ is composed of the classes with the fewest examples available across the four original datasets, which is convenient for evaluating the classification methods for images collected from multiple hospitals.

### TL strategies

The two TL strategies used in this study are described, and their differences are highlighted.

#### STL

STL is the most widely studied and spread strategy for computer vision tasks. When performing STL, two main stages can be identified [44]:

- Pre-training, which aims to acquire transferable knowledge from a source dataset $\mathcal {S}$.
- Adaptation, which leverages the knowledge acquired during pre-training to solve a task on a target dataset $\mathcal {T}$.

In the pre-training stage, $\mathcal {S}$ is divided into training $\mathcal {S}_{trn}$, validation $\mathcal {S}_{val}$, and test $\mathcal {S}_{tst}$ subsets. A randomly initialized neural network is then trained using batches $\mathcal {B}_{trn}$ sampled from $\mathcal {S}_{trn}$ and validated with batches $\mathcal {B}_{val}$ sampled from $\mathcal {S}_{val}$ to produce a pre-trained model. This process is commonly repeated with different hyperparameter configurations, yielding multiple pre-trained models. A single pre-trained model is subsequently selected based on its performance on the validation subset $\mathcal {S}_{val}$. In some cases, the selected pre-trained model is also evaluated on the test subset $\mathcal {S}_{tst}$.

In the adaptation stage, the target dataset $\mathcal {T}$ is typically divided into training $\mathcal {T}_{trn}$, validation $\mathcal {T}_{val}$, and test $\mathcal {T}_{tst}$ subsets. In order to transfer the knowledge acquired from the source dataset, a pre-trained neural network is first assembled: the feature extraction layers (also known as the backbone) are preserved with their original weights and biases, whereas the layers specific to the pre-training task are replaced with randomly initialized layers tailored to the target task. Then, the assembled neural network is trained using batches $\mathcal {B}_{trn}$ sampled from $\mathcal {T}_{trn}$ and validated with batches sampled from $\mathcal {T}_{val}$ to produce the model for the target task. Similar to the pre-training stage, multiple models can be produced with different hyperparameter configurations, from which a single model is selected based on its performance on the validation subset $\mathcal {T}_{val}$. Finally, the performance of the selected model is estimated using the test subset $\mathcal {T}_{tst}$.

Note that the pre-training and adaptation stages in STL have some distinctive characteristics.

- The classes in the source dataset $\mathcal {S}$ and the target dataset $\mathcal {T}$ are typically different; that is, the classes encountered during the adaptation stage are not seen during pre-training.
- Training is a batch-based iterative process, in which all classes within $\mathcal {S}$ are considered.
- Although the target dataset is smaller than the source dataset, it typically contains examples on the order of hundreds or even thousands per category.
- The STL performance is evaluated on a single task *T* that considers all classes and examples in $\mathcal {T}_{tst}$.

#### MTL

MTL is an alternative paradigm to STL that aims to obtain models that can adapt to novel tasks with unseen classes and very few examples per class [44]. In other words, MTL aims to achieve more efficient transfer in terms of data. Similar to STL, the transfer process of MTL consists of a pre-training stage followed by an adaptation stage. Although in MTL these stages are commonly referred to as meta-training and meta-test [45], for the sake of consistency and clarity, the terms pre-training and adaptation are used for both STL and MTL. This study focuses on two MTL formulations for classification: SFSC and GFSC.

In SFSC, the pre-training stage is equipped with meta-training $\mathcal {D}_{meta-trn}$ and meta-validation $\mathcal {D}_{meta-val}$ datasets, whereas the adaptation stage uses the meta-test $\mathcal {D}_{meta-tst}$ dataset. During the pre-training stage, an iterative training process is performed. In each iteration, the classification task $E_{meta-trn}$ is randomly generated. This task is known as an episode and is used to train the neural network. Each episode $E_{meta-trn}$ comprises a training $D_{trn}$ subset and a test $D_{tst}$ subset that share the same classes. To generate a meta-training episode $E_{meta-trn}$, *n* classes (known as *n*-way) are randomly selected from the set of meta-training classes $\mathcal {C}_{meta-trn}$. For each selected class, $k_{trn}$ and $k_{tst}$ examples are randomly sampled from $\mathcal {D}_{meta-trn}$ to form the $D_{trn}$ and $D_{tst}$ subsets. Typically, an episode is 5-way, and the number of samples per class is $k_{trn} = 5$ and $k_{tst} = 15$. Once the model is trained with a meta-training episode $E_{meta-trn}$, its performance is evaluated using an episode $E_{meta-val}$ sampled from the meta-validation set $\mathcal {D}_{meta-val}$. This pre-training process is referred to as episodic training.

In contrast to STL, the adaptation stage in SFSC follows an iterative process similar to pre-training, except that the meta-test episodes $E_{meta-tst}$ are sampled from $\mathcal {D}_{meta-tst}$. The performance of the model in the adaptation stage is estimated by averaging the performance scores over hundreds or thousands of episodes. Note that while STL focuses on assessing the capacity of the model to adapt to a single task *T*, comprising all examples and classes in the test subset of the target dataset, SFSC assesses the model’s capacity to adapt to a large number of small episodes $E_{meta-tst}$ sampled from $\mathcal {D}_{meta-tst}$. In other words, SFSC aims to estimate the adaptability of the model to tasks with novel classes and a few examples per class.

Finally, the difference between SFSC and GFSC lies in the classes and examples that constitute the $\mathcal {D}_{meta-val}$ and $\mathcal {D}_{meta-tst}$ sets. In SFSC, the set of classes for $\mathcal {D}_{meta-val}$ ($\mathcal {D}_{meta-tst}$) is equal to $\mathcal {C}_{meta-val}$ ($\mathcal {C}_{meta-tst}$), which is disjoint from the set of classes for $\mathcal {D}_{meta-trn}$. By contrast, in GFSL the set of classes for $\mathcal {D}_{meta-val}$ ($\mathcal {D}_{meta-tst}$) is equal to $\mathcal {C}_{meta-trn} \cup \mathcal {C}_{meta-val}$ ($\mathcal {C}_{meta-trn} \cup \mathcal {C}_{meta-tst}$). Thus, GFSC can be regarded as a generalization of SFSC in which evaluation episodes comprise not only unseen classes sampled from $\mathcal {C}_{meta-val}$ ($\mathcal {C}_{meta-tst}$) but also seen classes from $\mathcal {C}_{meta-trn}$.

### Experimental

A formulation of few-shot multi-label classification for chest X-rays is presented, along with TL and MTL methods, which are compared through empirical experiments.

#### Few-shot multi-label classification for chest X-rays

This study focuses on GFSC because this formulation allows modeling of common medical scenarios in which one seeks to classify opacities in an X-ray image associated with a combination of well-known pathologies and uncommon or even novel pathologies. Recall that in GFSC, a meta-validation or meta-test episode is composed of two types of classes. The first type comprises seen classes, which are used in meta-training episodes during the pre-training phase. In this context, the seen classes are regarded as known information even if examples have not been seen previously. The second type comprises unseen classes, which are completely new and appear only in meta-validation episodes during pre-training or meta-testing in the adaptation stage. These classes and examples are considered completely novel information. The greater the number of unseen classes, the more difficult the episode is owing to the higher amount of novel information, reaching a limit at the SFSC formulation (i.e., when all classes in the episode are unseen). However, in medical scenarios, an X-ray image presents opacities that are mostly expected to be associated with known pathologies, which contrasts with SFSC, where all pathologies are unknown.

**Fig. 3:**
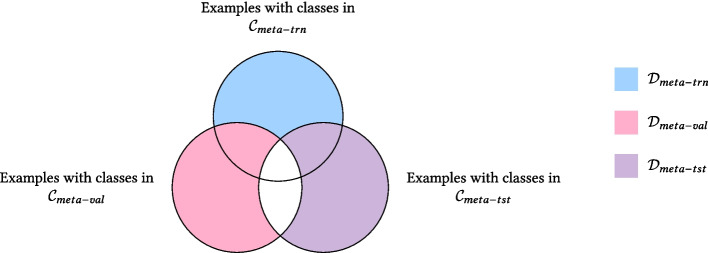
Venn diagram illustrating the relationship between meta-training $\mathcal {C}_{meta-trn}$, meta-validation $\mathcal {C}_{meta-val}$, and meta-test $\mathcal {C}_{meta-tst}$ class sets. Blue indicates examples in $\mathcal {D}_{meta-trn}$, pink in $\mathcal {D}_{meta-val}$, and purple in $\mathcal {D}_{meta-tst}$. Because these sets are disjoint and episodes are generated from only one set at a time, the meta-validation and the meta-test episodes contain examples that are not used during meta-training

To examine this, Algorithm 1, which generates multi-labeled episodes and allows control over the number of seen and unseen classes, as well as the minimum number of examples per class, is proposed. Owing to the multi-label nature of MetaChest, to generate episodes, the data were divided into $\mathcal {D}_{meta-trn}$, $\mathcal {D}_{meta-val}$, and $\mathcal {D}_{meta-tst}$ sets, as shown in Fig. 3. This division ensures that no examples are shared between the meta-training, meta-validation, and meta-test episodes, making the classification task more challenging and contributing to a more robust evaluation.

**Figure:**
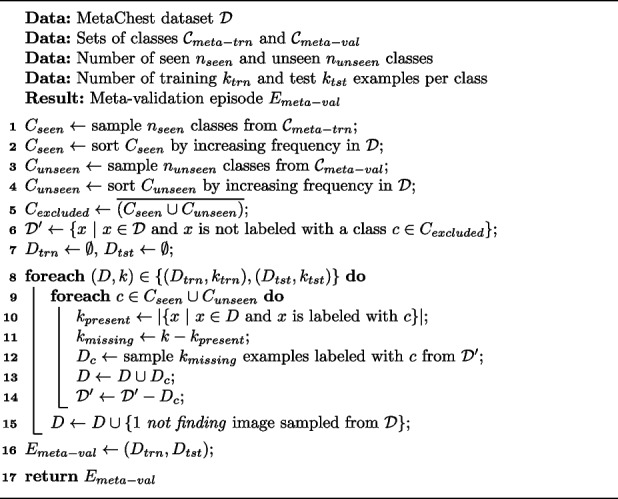
**Algorithm 1** Meta-validation episode generator

The pseudocode in Algorithm 1 outlines the meta-validation episode generation process, which can be applied similarly to the meta-test episodes. First, a set $C_{seen}$ of $n_{seen}$ classes is sampled from the meta-training classes $\mathcal {C}_{meta-trn}$ and sorted in ascending order according to their frequency in MetaChest (lines 1 and 2). Analogously, the set $C_{unseen}$ is sampled for the unseen classes (lines 3 and 4). Given the multi-label nature of the data, the set of excluded classes $C_{excluded}$ (line 5), which tracks classes that do not belong to $C_{seen}$ or $C_{unseen}$, is identified. Then, a sample set $\mathcal {D}'$ comprising examples *x* in MetaChest that are not labeled with any of the excluded classes $C_{excluded}$ (line 6) is generated, thereby avoiding the introduction of additional classes into the episodes. Next, the training subset $D_{trn}$ (lines 8–15) is generated. For each class *c* in ${(C_{seen} \cup C_{unseen})}$, the number of missing examples $k_{missing}$ in $D_{trn}$ needed to reach $k_{trn}$ (lines 9 and 11, respectively) is determined. Subsequently, a set $D_c$ with $k_{missing}$ examples from $\mathcal {D}'$ with class *c* is sampled and added to $D_{trn}$ (lines 12 and 13). Finally, a not finding X-ray example (line 15) is added to ensure that for every class, there is a negative example in $D_{trn}$, which enables the calculation of the receiver operating characteristic-based metrics used in this work. The test subset $D_{tst}$ is generated analogously.

#### Classification methods

Let the set of examples that are labeled with a class *c* in the training episode be denoted as $D_{c} = \{(\textbf{x}, \textbf{y}) \ | \ (\textbf{x}, \textbf{y}) \in D_{trn} \ \text {and} \ \textbf{y}[c] = 1 \}$, where $\textbf{x} \in \mathbb {R}^{h \times w \times 3}$ is a $h \times w$ image, and $\textbf{y} \in \{0, 1\}^{n-\text {way}}$ is the associated multi-label vector. Furthermore, let $f_{\phi }(\textbf{x}) \in \mathbb {R}^D$ denote the D-dimensional vector representation of $\textbf{x}$ computed by the backbone $f_{\phi }$ with trainable parameters $\phi$.

##### ProtoNet-ML

ProtoNet [31] is a multi-class classification method widely studied in the SFSC literature. In this study, an extension to handle multi-label classification, called ProtoNet-ML, is proposed. Following the original method, ProtoNet-ML computes a D-dimensional prototype $\textbf{z}_c \in \mathbb {R}^D$ for each class *c* as follows:

$$\begin{aligned} \textbf{z}_c = \frac{1}{|D_{c}|} \sum \limits _{(\textbf{x}_i, \textbf{y}_i) \in D_{c}}^{} f_{\phi }(\textbf{x}_i) \end{aligned}$$

The original multi-class ProtoNet estimates class probabilities by applying a softmax function over the negative distances between a test example and the class prototypes, implicitly associating the test example with the closest prototype. To enable associations with multiple prototypes, ProtoNet-ML introduces a transformation function over distances. Specifically, the transformation function $t: \mathbb {R}^D \times \mathbb {R}^D \rightarrow \mathbb {R}$ between a test example $(\textbf{x}, \textbf{y}) \in D_{tst}$ and the prototype $\textbf{z}_c$ for class *c* is defined as

$$\begin{aligned} t(f_{\phi }(\textbf{x}), \textbf{z}_c) = \mu _c - d(f_{\phi }(\textbf{x}), \textbf{z}_c) \end{aligned}$$

where $d(f_{\phi }(\textbf{x}), \textbf{z}_c) = || f_{\phi }(\textbf{x}) - \textbf{z}_c ||$ is the Euclidean distance, and $\mu _c$ is the mean distance between the prototype for the class *c* and all training examples in the episode, i.e.,

$$\begin{aligned} \mu _c = \frac{1}{\vert D_{trn} \vert } \sum \limits _{(\textbf{x}_i, \textbf{y}_i) \in D_{trn}} d(f_{\phi }(\textbf{x}_{i}), \textbf{z}_c) \end{aligned}$$

**Fig. 4:**
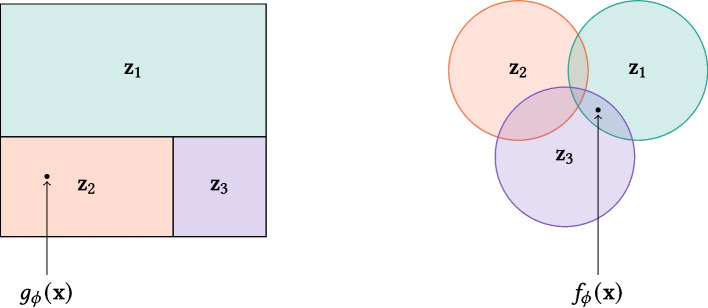
Representation subspaces for multi-class ProtoNet (left) and ProtoNet-ML (right). In multi-class ProtoNet, a representation vector $g_{\phi }(\textbf{x})$ is associated with only one prototype, whereas in ProtoNet-ML, a representation vector $f_{\phi }(\textbf{x})$ may be associated with one or more prototypes. Note that $f_{\phi }(\cdot )$ is the network backbone followed by an encoding layer to reduce representation vector dimensionality

Subtracting the example-prototype distance from the mean distance maps, examples closer than the mean are mapped to increasingly positive values, whereas those farther away are mapped to increasingly negative values. This transformation can be used to compute a probability distribution for a test example $\textbf{x}$ belonging to class *c* as follows:

$$\begin{aligned} p(\textbf{y}[c] = 1 \mid \textbf{x}) = \sigma (t(f_{\phi }(\textbf{x}), \textbf{z}_c)) \end{aligned}$$

where $\sigma$ denotes the sigmoid function. Unlike multi-class prototypes, which partition the D-dimensional representation space into disjoint subspaces, multi-label prototypes correspond to subspaces that may overlap. This allows the representation of a single example to fall into more than one subspace simultaneously, as shown in Fig. 4.

Beyond the Euclidean distance, ProtoNet-ML can be instantiated with other functions, including the Minkowski distance and, with slight modifications, the cosine distance. Moreover, ProtoNet-ML is a flexible method that supports arbitrary activation functions and can operate directly on logits. However, in the experiments, the sigmoid function was employed because it is the conventional and most natural choice for binary classification.

##### BatchBased

BatchBased is a method inspired by ref. [46] that employs STL-based training while maintaining MTL-based episode evaluation. On top of the backbone $f_{\phi }$, BatchBased adds a head module $g_{\varphi }$ (a single fully connected layer) with trainable parameters $\varphi$. The probability distribution for a test example $\textbf{x}$ given a class *c* is computed as:

$$\begin{aligned} p(\textbf{y}[c] = 1 \mid \textbf{x}) = \sigma (g_{\varphi }(f_{\phi }(\textbf{x}))) \end{aligned}$$

As in STL, the learning process is conducted in epochs, during which the input data are fed to the model in batches. Note that STL batches are constructed from all classes in $\mathcal {C}_{meta-trn}$, whereas MTL episodes involve only a subset of these classes. The model parameters $\phi$ and $\varphi$ are updated for each batch by backpropagating through the entire network. After each epoch is completed, an episode-based evaluation is conducted using the MTL paradigm. Specifically, for each $E_{meta-val}$ or $E_{meta-tst}$ episode, the $f_{\phi }$ parameters are frozen, whereas the head module $g_{\varphi }$ is replaced and updated in $D_{trn}$. To update the head parameters $\varphi$, an iterative process is repeated $t_{steps}$ steps. At each step, a subset of examples *M* is randomly sampled from $D_{trn}$, where $\vert M\vert$ is the proportion $ptc_{trn}$ of $D_{trn}$. Subsequently, the head parameters $\varphi$ are updated with a learning rate $lr_{head}$ via backpropagation using *M*. Here, $t_{steps}$, $ptc_{trn}$, and $lr_{head}$ are considered hyperparameters.

## Results and Discussion

In this section, the adaptation process of different models across various formulations of the multi-label classification task is analyzed using a few examples for each pathology. First, the manner in which two distinct learning paradigms leverage ImageNet pre-training is compared. Next, their behaviors across a broad set of few-shot learning tasks designed to reflect the challenges commonly encountered in medical settings are examined. In addition, factors influencing the adaptation process, including image resolution and variations in architectural connectivity patterns, are investigated. Finally, the influence of hyperparameters on the classification performance is examined, and illustrative examples of the resulting model predictions are provided.

### Experimental setup

In the empirical evaluation, certain training and method hyperparameters are fixed, whereas others are varied to assess their impact on the classification performance. The experimental setup is described in detail below. The code used to reproduce the main findings is publicly available at https://github.com/bereml/meta-cxr.

#### Training

The default hyperparameter configurations are presented in Table 3. Unless otherwise specified, the reported results correspond to the BatchBased configuration.

#### Evaluation

The model performance is measured as the average of over 10,000 episodes sampled from the meta-test set. As is common in GFSL [47], the seen and unseen classes are separately evaluated, and the harmonic mean (HM) of their scores is reported. However, area under the curve-receiver operating characteristic (AUC-ROC) is adopted instead of accuracy [48, 49] to align with evaluation standards in the medical domain.

Three metrics commonly used in GFSL [47] are employed, each reported with a 95% CI: one computed for the seen classes, another for the unseen classes, and the third for the HM, defined as follows:

- *Seen*: The AUC-ROC of all labels of the seen classes in the episode as a single binary classification task.
- *Unseen*: The AUC-ROC of all labels of the unseen classes in the episode as a single binary classification task.
- *HM*: The harmonic mean of *Seen* and *Unseen*, i.e.:
  $$\begin{aligned} HM = \frac{2 \ \times \ Seen \ \times \ Unseen}{Seen \ + \ Unseen} \end{aligned}$$
  The harmonic mean is commonly used in GFSL because it mitigates the dominance of seen classes in the overall performance [47].

### Leveraging ImageNet

The comparison between BatchBased and ProtoNet-ML begins using models that are randomly initialized and pre-trained on either ImageNet-1K or ImageNet-21K. This experiment is performed using the MobileNetV3Large100 architecture, as it is the only pre-trained model available for both versions of ImageNet.

**Table 3:** Default hyperparameter configurations

Parameter	Configuration
*Data*	
Distribution	Complete
Image size	384
*Task*	
Training batch size	64
Training *n*-way, $k_{trn}$ $k_{tst}$	3, 30, 30
Validation *n*-way, $k_{trn}$ $k_{tst}$	3, 30, 30
Test *n*-way, $k_{trn}$ $k_{tst}$	3, 30, 30
*Backbone*	
Architecture	MobileNetV3Small075
Pre-training	I1K
*Training*	
Meta-trn, meta-val, meta-tst episodes	1,000, 100, 10,000
Max epochs	150
Optimizer	AdamW
Stop metric, patience	*HM*, 10
Float precision	16bit
*BatchBased*	
Meta-trn LR	0.0001
Meta-val $t_{steps}$, $ptc_{trn}$, $lr_{head}$	100, 0.5, 0.05
Meta-tst $t_{steps}$, $ptc_{trn}$, $lr_{head}$	100, 0.5, 0.05
*ProtoNet-ML*	
Encoding layer type, size	Average pooling, 128
Meta-training LR	0.0001

As shown in Table 4, BatchBased consistently outperforms ProtoNet-ML across all models and metrics. For instance, on ImageNet-1K, BatchBased surpasses ProtoNet-ML by 4.31 *HM* points. When comparing the ImageNet-1K and ImageNet-21K models for BatchBased, the former achieves better results across all metrics. For example, ImageNet-1K yields an improvement of 0.78 *HM* points compared with ImageNet-21K. Furthermore, BatchBased initialization with ImageNet-21K weights demonstrates a 4.47 *HM* points gain over randomly initialized models. The literature on few-example regimes in inter-domain scenarios reports inconclusive findings regarding the benefits of using pre-trained models on ImageNet-1K [3]. However, these results indicate that using pre-trained models consistently improves the performance on chest X-ray images.

**Table 4:** Comparison of randomly initialized and ImageNet-pre-trained MobileNetV3Large100 models for BatchBased and ProtoNet-ML

Model	*Seen* $\uparrow$	*Unseen* $\uparrow$	*HM* $\uparrow$
*BatchBased*			
Random	82.42 ± 0.14	78.17 ± 0.35	78.83 ± 0.25
ImageNet-1K	**86.49 ± 0.11**	**83.80 ± 0.31**	**84.08 ± 0.22**
ImageNet-21K	85.89 ± 0.12	82.98 ± 0.32	83.30 ± 0.22
*ProtoNet-ML*			
Random	76.48 ± 0.14	75.69 ± 0.34	74.83 ± 0.23
ImageNet-1K	82.10 ± 0.12	79.45 ± 0.30	79.77 ± 0.20
ImageNet-21K	81.89 ± 0.12	80.18 ± 0.31	80.06 ± 0.21

The values in bold indicate the best performance

### Few-shot learning *vs* TL

**Fig. 5:**
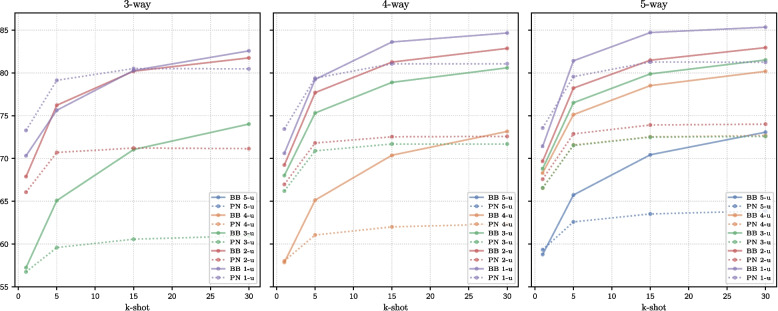
Performance of BatchBased (BB) and ProtoNet-ML (PN) on pathology classification tasks across varying *n*-way, *n*-unseen, and *k*-shot configurations

Building on the results from the previous subsection, the different aspects inherent in few-shot classification for BatchBased and ProtoNet-ML are examined. Table 5 compares the results of both methods across different task configurations, and Fig. 5 illustrates the behavioral trends of each method.

**Table 5:** Comparison of BatchBased and ProtoNet-ML on pathology classification tasks evaluated with harmonic mean

*k*-shot	3-way		4-way		5-way	
	BatchBased	ProtoNet-ML	BatchBased	ProtoNet-ML	BatchBased	ProtoNet-ML
1-unseen						
1	70.32 ± 0.31	**73.28 ± 0.21**	70.61 ± 0.27	**73.44 ± 0.17**	71.42 ± 0.24	**73.56 ± 0.15**
5	75.63 ± 0.29	**79.13 ± 0.18**	79.23 ± 0.20	**79.38 ± 0.13**	**81.41 ± 0.15**	79.56 ± 0.11
15	80.28 ± 0.26	**80.51 ± 0.19**	**83.61 ± 0.14**	81.06 ± 0.12	**84.71 ± 0.10**	81.27 ± 0.10
30	**82.57 ± 0.23**	80.47 ± 0.20	**84.66 ± 0.12**	81.06 ± 0.12	**85.34 ± 0.08**	81.24 ± 0.10
2-unseen						
1	**67.89 ± 0.20**	66.06 ± 0.15	**69.24 ± 0.15**	66.97 ± 0.13	**69.68 ± 0.13**	67.58 ± 0.12
5	**76.22 ± 0.15**	70.70 ± 0.11	**77.69 ± 0.10**	71.81 ± 0.09	**78.22 ± 0.09**	72.87 ± 0.08
15	**80.20 ± 0.14**	71.22 ± 0.11	**81.26 ± 0.09**	72.53 ± 0.08	**81.48 ± 0.07**	73.91 ± 0.07
30	**81.75 ± 0.13**	71.15 ± 0.11	**82.86 ± 0.08**	72.58 ± 0.08	**82.95 ± 0.07**	74.01 ± 0.07
3-unseen						
1	**57.25 ± 0.12**	56.75 ± 0.10	**68.01 ± 0.15**	66.20 ± 0.13	**68.81 ± 0.12**	66.56 ± 0.11
5	**65.08 ± 0.11**	59.59 ± 0.09	**75.31 ± 0.11**	70.89 ± 0.09	**76.51 ± 0.07**	71.52 ± 0.07
15	**71.04 ± 0.09**	60.57 ± 0.08	**78.89 ± 0.10**	71.69 ± 0.08	**79.88 ± 0.06**	72.49 ± 0.07
30	**74.02 ± 0.08**	60.96 ± 0.08	**80.59 ± 0.10**	71.69 ± 0.08	**81.51 ± 0.06**	72.57 ± 0.07
4-unseen						
1			57.86 ± 0.10	**58.03 ± 0.08**	**68.30 ± 0.13**	66.55 ± 0.12
5			**65.13 ± 0.08**	61.06 ± 0.07	**75.12 ± 0.09**	71.57 ± 0.07
15			**70.38 ± 0.07**	62.01 ± 0.06	**78.51 ± 0.08**	72.53 ± 0.07
30			**73.16 ± 0.06**	62.35 ± 0.06	**80.18 ± 0.08**	72.68 ± 0.07
5-unseen						
1					58.79 ± 0.09	**59.34 ± 0.07**
5					**65.73 ± 0.07**	62.59 ± 0.05
15					**70.43 ± 0.05**	63.52 ± 0.04
30					**73.07 ± 0.05**	63.87 ± 0.04

The values in bold indicate the best HM performance for each task and method

As observed, ProtoNet-ML achieves an improved performance in only a limited subset of task configurations. Table 5 shows that these improvements occur primarily in the 1-unseen setting and tend to disappear as the number of shots (*k*-shot) or classes (*n*-way) increases. In the remaining task configurations, BatchBased outperforms ProtoNet-ML. Moreover, as shown in Fig. 5, BatchBased demonstrates improved performance as the number of shots increases, whereas ProtoNet-ML’s performance remains nearly constant. These findings are consistent with those reported by Luo et al. [50], who compared the performance of conventional models with MTL methods, such as ProtoNet on SFSC tasks across various natural image datasets. They found that conventional models tend to scale better than MTL approaches, particularly for fine-grained datasets. In medical applications, where datasets often include many classes and dozens of examples per class, these results suggest that BatchBased is a more effective approach for training pathology classifiers.

### Pathology classification complexity

**Fig. 6:**
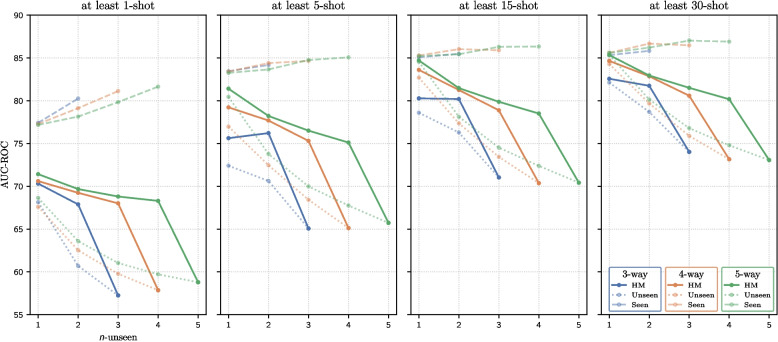
Performance on pathology classification tasks with few examples, varying the number of classes (*n*-way) from 3 to 5, the number of unseen classes (*n*-unseen) from 1 to *n*-way, and the number of training shots per class ($k_{trn}$) to at least 5, 10, 15, and 30. Solid lines indicate the harmonic mean (HM), dashed lines indicate the area under the curve-receiver operating characteristic (AUC-ROC) for seen classes, and dotted lines indicate the AUC-ROC for unseen classes

The effectiveness of BatchBased is evaluated by varying the number of classes per episode, the number of unseen classes, and the number of examples per class. The results of these experiments are presented in Table 6 and the corresponding performance trends are illustrated in Fig. 6.

**Table 6:** Seen, unseen, and HM metrics for pathology classification tasks across *n*-way, *n*-unseen, and $k_{trn}$ configurations

*n*-unseen	**1-shot**			**5-shot**		
	*Seen* $\uparrow$	*Unseen* $\uparrow$	*HM* $\uparrow$	*Seen* $\uparrow$	*Unseen* $\uparrow$	*HM* $\uparrow$
3-way						
1	77.42 ± 0.22	68.15 ± 0.41	70.32 ± 0.31	83.45 ± 0.14	72.41 ± 0.40	75.63 ± 0.29
2	80.26 ± 0.30	60.70 ± 0.20	67.89 ± 0.20	84.18 ± 0.21	70.63 ± 0.17	76.22 ± 0.15
3		57.25 ± 0.12	57.25 ± 0.12		65.08 ± 0.11	65.08 ± 0.11
4-way						
1	77.29 ± 0.17	67.60 ± 0.36	70.61 ± 0.27	83.41 ± 0.11	76.98 ± 0.29	79.23 ± 0.20
2	79.13 ± 0.17	62.53 ± 0.19	69.24 ± 0.15	84.40 ± 0.10	72.47 ± 0.15	77.69 ± 0.10
3	81.13 ± 0.27	59.78 ± 0.14	68.01 ± 0.15	84.66 ± 0.19	68.43 ± 0.11	75.31 ± 0.11
4		57.86 ± 0.10	57.86 ± 0.10		65.13 ± 0.08	65.13 ± 0.08
5-way						
1	77.18 ± 0.14	68.65 ± 0.34	71.42 ± 0.24	83.26 ± 0.10	80.46 ± 0.22	81.41 ± 0.15
2	78.15 ± 0.14	63.60 ± 0.18	69.68 ± 0.13	83.66 ± 0.08	73.78 ± 0.13	78.22 ± 0.09
3	79.84 ± 0.16	61.04 ± 0.14	68.81 ± 0.12	84.76 ± 0.09	69.99 ± 0.10	76.51 ± 0.07
4	81.65 ± 0.25	59.72 ± 0.12	68.30 ± 0.13	85.07 ± 0.18	67.76 ± 0.08	75.12 ± 0.09
5		58.79 ± 0.09	58.79 ± 0.09		65.73 ± 0.07	65.73 ± 0.07
*n*-unseen	**15-shot**			**30-shot**		
	*Seen* $\uparrow$	*Unseen* $\uparrow$	*HM* $\uparrow$	*Seen* $\uparrow$	*Unseen* $\uparrow$	*HM* $\uparrow$
3-way						
1	85.08 ± 0.12	78.60 ± 0.36	80.28 ± 0.26	85.33 ± 0.12	82.13 ± 0.32	82.57 ± 0.23
2	85.48 ± 0.19	76.29 ± 0.15	80.20 ± 0.14	85.83 ± 0.19	78.70 ± 0.14	81.75 ± 0.13
3		71.04 ± 0.09	71.04 ± 0.09		74.02 ± 0.08	74.02 ± 0.08
4-way						
1	85.29 ± 0.09	82.71 ± 0.21	83.61 ± 0.14	85.60 ± 0.08	84.30 ± 0.18	84.66 ± 0.12
2	86.03 ± 0.09	77.36 ± 0.13	81.26 ± 0.09	86.68 ± 0.09	79.69 ± 0.12	82.86 ± 0.08
3	85.90 ± 0.18	73.44 ± 0.09	78.89 ± 0.10	86.48 ± 0.17	75.90 ± 0.08	80.59 ± 0.10
4		70.38 ± 0.07	70.38 ± 0.07		73.16 ± 0.06	73.16 ± 0.06
5-way						
1	85.22 ± 0.08	84.56 ± 0.15	84.71 ± 0.10	85.59 ± 0.07	85.36 ± 0.13	85.34 ± 0.08
2	85.44 ± 0.07	78.13 ± 0.11	81.48 ± 0.07	86.21 ± 0.06	80.15 ± 0.11	82.95 ± 0.07
3	86.30 ± 0.08	74.52 ± 0.08	79.88 ± 0.06	87.03 ± 0.08	76.80 ± 0.08	81.51 ± 0.06
4	86.34 ± 0.16	72.39 ± 0.07	78.51 ± 0.08	86.91 ± 0.16	74.79 ± 0.06	80.18 ± 0.08
5		70.43 ± 0.05	70.43 ± 0.05		73.07 ± 0.05	73.07 ± 0.05

#### Classes per episode *n*-way

As noted, as the number of classes in the episode increases while the number of unseen classes remains constant, the performance improves consistently. For example, in Table 6, the 5-way, 3-unseen configuration with 15-shot configuration outperforms the 4-way, 3-unseen configuration with the same number of shots by 0.99 *HM* points, highlighting the performance gain from adding a single class. As shown in Fig. 6, the 4-way configurations (solid orange line) consistently achieved higher *HM* scores than the 3-way configurations (solid blue line), whereas the 5-way configurations (solid green line) outperforms the 4-way configurations (solid orange line). This suggests that increasing the number of classes per episode, and consequently the number of examples, reduces the task complexity and leads to improved performance. Similar results have been reported in the SFSC literature on natural image datasets, where a higher number of classes per episode consistently improves the classification performance [50].

#### Unseen classes *n*-unseen

As the number of unseen classes increases the performance degrades, as illustrated by the downward trends in the *HM* curves in Fig. 6. From a learning paradigm perspective, this allows the analysis of the complexity of episodes when transitioning from a GFSL formulation (less novel information) to a SFSL formulation (entirely novel information). Notably, the performance falls in the experiments was considerably larger when transitioning from GFSL (with at least one seen class) to SFSL (with all classes being unseen).

#### Examples per class $k_{trn}$

As reported, the performance steadily improves as the number of examples per class increases. This trend is clearly illustrated in Fig. 6, which reveals a progressive improvement in performance across subfigures corresponding to at least 1, 5, 15, and 30 shots per class. For example, in Table 6, under the 5-way 1-unseen configuration, the performance improves by 9.99, 13.29, and 13.92 *HM* points for $k_{trn} = 5$, $k_{trn} = 15$, and $k_{trn} = 30$, respectively, compared to $k_{trn} = 1$. This suggests that increasing the number of examples reduces task complexity, thereby enabling the model to achieve a higher performance.

#### Confidence interval

As viewed in the results, an increase in the number of classes consistently results in narrower confidence intervals. Similarly, increasing the number of examples per class yields narrower intervals for both seen and unseen classes.

### X-ray resolution

In most cases, deep neural networks used for natural image classification are trained on low-resolution images (typically $224\times 224$ or $256\times 256$ pixels) to reduce computational cost. Such resolutions are adequate for datasets such as ImageNet, which involve coarse-grained classification tasks characterized by visually distinct categories (e.g., cars and dogs). Even in few-shot classification tasks on mini-ImageNet, a resolution of 64 and shallow architectures (typically 4 to 6 layers) are commonly used, helping to mitigate the parameter explosion.

By contrast, classifying pathologies on chest X-rays is a fine-grained task because the opacity patterns that distinguish different pathologies are often extremely subtle. The literature on the effect of resolution is limited, particularly in the context of few-shot classification. Consequently, determining the most appropriate resolution for pathology classification of chest X-rays remains an important open research question.

In this experiment, the models are trained using three different architectures, and the X-ray resolution is progressively incremented to study their effects. The images are resized using the Lanczos algorithm, a high-quality resampling method known for preserving edge sharpness and fine details [51]. The results are summarized in Table 7.

**Table 7:** Comparison of MobileNetV3-Small-0.75, ConvNeXt-Tiny, and DenseNet-121 models on chest X-rays at varying input resolutions ($224\times 224$, $384\times384$, $512\times 512$, $768\times 768$, and $1024\times 1024$ pixels)

Resolution	*Seen* $\uparrow$	*Unseen* $\uparrow$	*HM* $\uparrow$
*MobileNetV3-Small-0.75*			
224	84.29 ± 0.13	81.75 ± 0.32	81.87 ± 0.23
384	85.73 ± 0.12	82.61 ± 0.32	83.03 ± 0.22
512	85.89 ± 0.12	82.53 ± 0.32	83.06 ± 0.22
768	**86.27 ± 0.11**	**82.92 ± 0.31**	**83.49 ± 0.22**
1024	86.39 ± 0.11	82.54 ± 0.32	83.23 ± 0.23
*ConvNext-Tiny*			
224	87.22 ± 0.11	84.50 ± 0.30	84.88 ± 0.21
384	87.85 ± 0.10	84.58 ± 0.30	85.22 ± 0.21
512	88.09 ± 0.10	84.44 ± 0.30	85.24 ± 0.21
768	**88.16 ± 0.10**	**84.53 ± 0.30**	**85.29 ± 0.22**
*DenseNet-121*			
224	84.97 ± 0.12	83.27 ± 0.29	83.17 ± 0.21
384	85.04 ± 0.12	82.97 ± 0.29	83.03 ± 0.20
512	**85.39 ± 0.12**	**83.37 ± 0.29**	**83.43 ± 0.20**

A fixed batch size of 32 was used to ensure a fair comparison across architectures. The values in bold indicate the best performance for each configuration

All three architectures exhibit improved performance at a resolution of $384\times 384$, which is higher than the resolution commonly used in ImageNet. For instance, MobileNetV3-Small-0.75 improves by 1.16 *HM* points, whereas ConvNeXt-Tiny and DenseNet-121 achieve gains of 0.34 and 0.14 *HM* points, respectively.

These results are consistent with previous findings in the medical imaging literature under a complete data regime. For example, in mammography, lesions are detected more accurately in images with a resolution of $1700 \times 2100$ pixels [52]. Similarly, in the case of chest X-rays, Rochmawanti and Utaminingrum [53] compared the performance of two models on the ChestX-ray14 dataset at resolutions of $64\times 64$ and $320\times 320$ pixels, observing improved performance at a higher resolution.

**Fig. 7:**
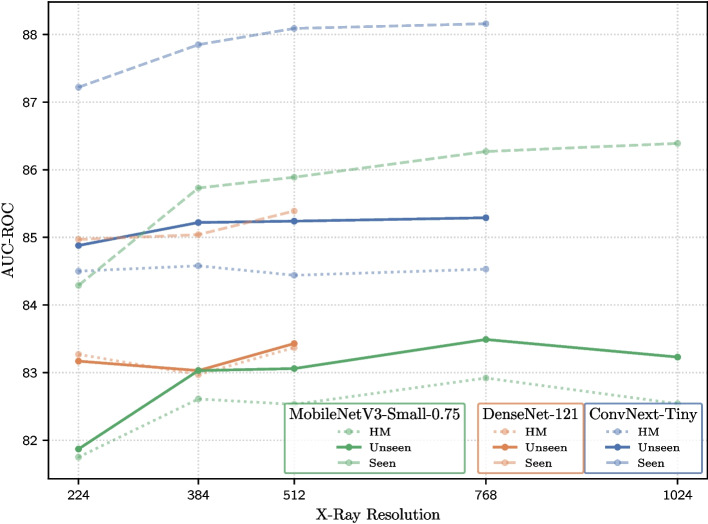
Comparison of convolutional architectures across progressively increasing chest X-ray resolutions

For MobileNetV3-Small-0.75, the performance progressively improves as the resolution increases to $768\times 768$ but begins to decline at higher resolutions, as shown in Fig. 7. By contrast, both ConvNeXt-Tiny and DenseNet-121 exhibit consistent improvements with increasing resolution. ConvNeXt-Tiny outperforms the other two architectures across all evaluated resolutions. The highest performance is achieved with this architecture at a resolution of $768\times 768$, although it surpass the best result of MobileNetV3-Small-0.75 by only 1.8 *HM* points. ConvNeXt-Tiny improves upon MobileNetV3-Small-0.75 at the default resolution used in this study ($384\times 384$) by only 2.26 *HM* points.

This finding is particularly relevant because increasing the image resolution substantially affects the memory requirements and computational costs for both training and inference. Furthermore, this also impacts the memory required for intermediate computations, gradients, and activations within the neural network, thereby making high-resolution training substantially more demanding. Computational cost also increases sharply, as higher resolutions require more multiply-accumulate operations in each layer. In addition, GPU memory usage grows, limiting batch sizes and potentially slowing training. Model complexity further interacts with image resolution: deeper or wider architectures may struggle to efficiently process very high-resolution inputs without optimization strategies such as mixed precision. Owing to these constraints, certain experiments could not be performed. For example, training ConvNeXt-Tiny at $1024 \times 1024$ and DenseNet-121 at $768 \times 768$ and $1024 \times 1024$ was not feasible owing to GPU memory limitations and high computational costs, highlighting a practical limitation in scaling experiments to very high-resolution images.

### Architectures

The manner in which the connectivity patterns and the number of parameters/operations influence the pathology classification performance is investigated. This is particularly relevant because evidence from the complete data regime in language modeling [54, 55] and computer vision [18, 56] suggests that increasing the network size and training data consistently reduces error. For MTL, Chen et al. [46] evaluated several convolutional architectures for few-shot multi-class classification on mini-ImageNet and a reduced version of the CUB dataset [57]. Their findings were inconclusive: while deeper architectures improved the CUB performance, gains on mini-ImageNet were observed only in certain cases.

In this experiment, the effects of convolutional and attention-based connectivity patterns are investigated using popular vision architectures. For both types, efficient architectures are examined with relatively few parameters and operations, as well as larger networks. For efficient convolutional architectures, the focus is on ConvNeXt-Atto [58] and lightweight versions of MobileNet [59], whereas for the Transformer-based models, MobileViTV2-1.0 [60] is used. For larger convolutional architectures, DenseNet-121, DenseNet-161 [61], and ConvNeXt-Tiny are evaluated, and for Transformer-based models, MobileViTV2-2.0 [60] is considered. Table 8 summarizes the results, comparing the performance of these architectures along with their number of parameters and operations.

**Table 8:** Comparison of convolutional and Transformer-based vision architectures

Architecture	Type	Parameter (M)$\downarrow$	MACs (G)$\downarrow$	$Seen\uparrow$	$Unseen\uparrow$	$HM\uparrow$
*Efficient*						
MobileNetV3-Small-075	Conv	1.01	0.11	85.73 ± 0.12	82.61 ± 0.32	83.03 ± 0.22
MobileNetV3-Large-1.0	Conv	4.20	0.62	86.75 ± 0.11	84.01 ± 0.30	84.37 ± 0.21
MobileViTV2-1.0	Tsfm	4.38	4.06	86.13 ± 0.11	82.47 ± 0.30	83.21 ± 0.21
ConvNext-Atto	Conv	3.37	1.61	86.88 ± 0.11	84.47 ± 0.30	84.71 ± 0.21
*Large*						
DenseNet-121	Conv	6.94	8.09	85.04 ± 0.12	82.97 ± 0.29	83.03 ± 0.20
DenseNet-161	Conv	26.46	22.36	86.22 ± 0.11	83.46 ± 0.29	83.90 ± 0.20
ConvNext-Tiny	Conv	27.81	18.36	**87.85 ± 0.10**	**84.58 ± 0.30**	**85.22 ± 0.21**
MobileViTV2-2.0	Tsfm	17.42	16.07	87.15 ± 0.11	84.32 ± 0.30	84.75 ± 0.21

Models are grouped into two categories based on the number of parameters: efficient and large

The values in bold indicate the best performance

Among the large architectures, ConvNeXt-Tiny achieves the highest performance, reaching 85.22 *HM* points. Notably, it outperforms DenseNet-161 by 1.32 *HM* points, an architecture previously shown to be effective for medical image analysis [12, 53, 62, 63].

Among the efficient architectures, ConvNeXt-Atto achieves the highest performance with 84.71 *HM* points, followed by MobileNetV3-Large-1.0. Compared with the default architecture used in this study (MobileNetV3-Small-0.75), ConvNeXt-Atto offers an improvement of only 1.68 *HM* points. However, MobileNetV3-Small-0.75 requires only 29.97% of the parameters and 6.83% of the computational operations used by ConvNeXt-Atto. This substantial reduction in resource requirements makes it particularly well-suited for deployment in resource-constrained environments such as on-device medical image analysis systems.

**Fig. 8:**
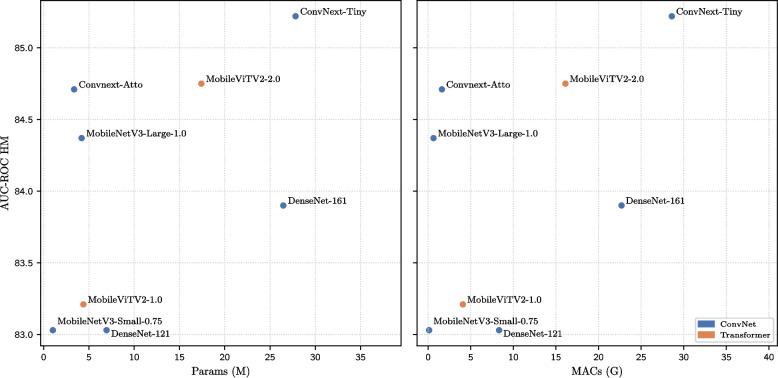
Trade-off between classification performance and the number of parameters or multiply-accumulate operations (MACs) for different convolutional and Transformer architectures

Interestingly, across both efficient and large architectures, convolutional models outperforms their Transformer-based counterparts. This trend is illustrated in Fig. 8, which depicts the relationship between the model performance and computational efficiency for the evaluated architectures.

Efficient neural network architectures reduce the computational requirements without significantly compromising the performance, thus offering several practical benefits. These architectures can run on devices with limited hardware resources, lowering costs, and expanding accessibility. Moreover, their efficiency can enable scalable deployment and seamless integration into existing medical infrastructure, including those in remote or resource-constrained regions.

### Hyperparameter analysis

The impact of different hyperparameter configurations on the method performance is assessed by varying the selected hyperparameters and evaluating the resulting classification outcomes. For the BatchBased method, the two hyperparameters are tuned during the adaptation phase. The first is Meta-tst $lr_{head}$, the learning rate used to update head parameters $\varphi$. The second is Meta-tst $ptc_{trn}$, the proportion of examples incorporated into the training steps for each episode $E_{meta-tst}$. For ProtoNet-ML, two types of encoding layers are investigated across different output sizes: a fully connected layer and an average pooling layer. The results of the experiments are presented in Table 9. For BatchBased, a lower learning rate of 0.005 consistently yields the best results, regardless of the chosen $ptc_{trn}$ value. Among the three evaluated configurations of $ptc_{trn}$, the best performance is achieved with a value of 0.5. By contrast, ProtoNet-ML obtains the best performance when using an average pooling layer with an output size of 128. Overall, BatchBased shows low sensitivity to hyperparameter variations, maintaining consistent performance across configurations, thus underscoring its robustness.

**Table 9:** Comparison of model performance across BatchBased and ProtoNet-ML hyperparameter configurations

Hyperparameter	*Seen* $\uparrow$	*Unseen* $\uparrow$	*HM* $\uparrow$
*BatchBased*			
*Meta-tst* $lr_{head}=0.01$,$ptc_{trn}=$			
0.25	85.07 ± 0.12	81.50 ± 0.33	82.01 ± 0.24
0.5	85.12 ± 0.12	81.55 ± 0.33	82.07 ± 0.23
0.75	85.11 ± 0.12	81.55 ± 0.33	82.07 ± 0.23
*Meta-tst* $lr_{head}=0.005$,$ptc_{trn}=$			
0.25	85.34 ± 0.12	82.15 ± 0.32	82.54 ± 0.23
0.5	**85.33 **± **0.12**	**82.13 **± **0.32**	**82.57 **± **0.23**
0.75	85.32 ± 0.12	82.16 ± 0.32	82.54 ± 0.23
*ProtoNet-ML*			
*Average pooling*			
96	80.16 ± 0.14	79.02 ± 0.36	78.17 ± 0.25
128	**81.88 **± **0.12**	**80.95 **± **0.30**	**80.47 **± **0.20**
144	80.61 ± 0.15	77.90 ± 0.37	77.70 ± 0.27
*Fully connected layer*			
96	80.81 ± 0.14	79.06 ± 0.36	78.51 ± 0.25
128	82.05 ± 0.15	76.20 ± 0.38	77.44 ± 0.27
144	81.12 ± 0.14	77.46 ± 0.37	77.76 ± 0.26

The values in bold indicate the best performance for each configuration

### Visualization of model predictions

**Fig. 9:**
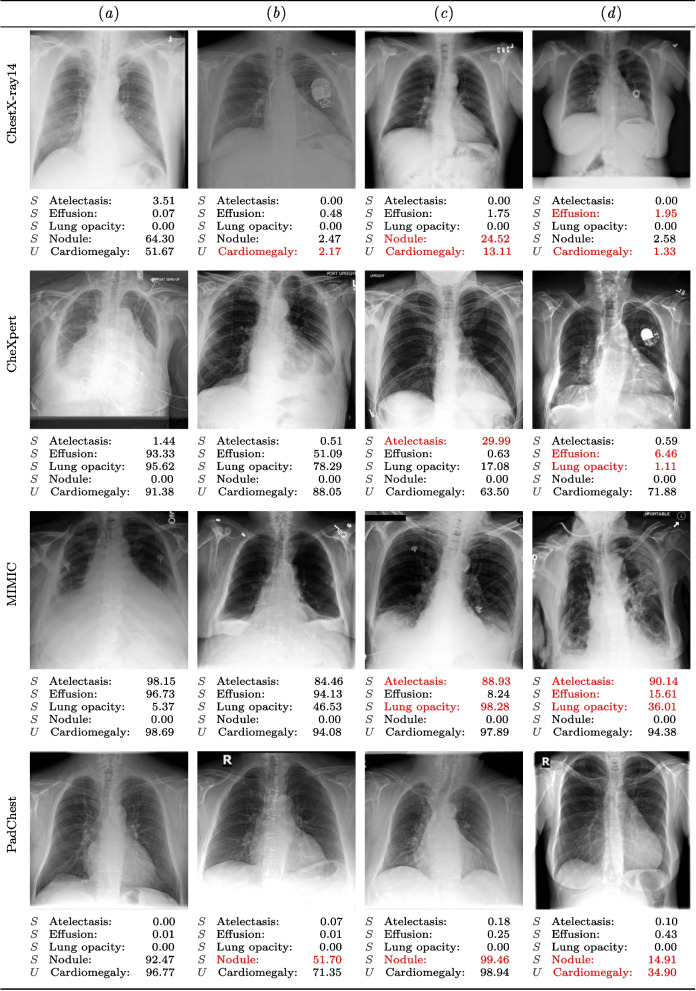
X-ray examples with predicted labels for the 5-way, 1-unseen classification task with 30-shots per class. Each row shows four examples per dataset in MetaChest. Labels below each chest X-ray are annotated with *S* and *U* for seen and unseen classes, respectively. Numbers indicate predicted class probabilities; incorrect predictions are shown in red

A set of model predictions are visualized to further examine its behavior qualitatively. Figure 9 presents selected chest X-ray examples along with their corresponding predictions across the four datasets comprising MetaChest. The examples are arranged from left to right, progressing from correctly classified cases to those with substantial prediction errors. For example, in the last row, the image in column (*a*) shows a PadChest X-ray for which the model correctly predicts all four seen classes, as well as the unseen class. By contrast, the image in column (*d*) of the same row illustrates a case in which the model correctly identifies three categories but misclassifies two pathologies, one seen and one unseen, both in red.

## Conclusions

This study investigated the key factors influencing model training for pathology classification of chest X-rays under few-shot scenarios. To this end, MetaChest, a benchmark dataset that integrates four publicly available chest X-ray datasets, was introduced. MetaChest provides a data partition specifically designed for MTL assessment, along with a novel multi-label episode generation algorithm. Using MetaChest, a family of diverse classification tasks were generated to compare two representative learning methods: one based on STL and the other widely adopted in SFSC. How various task-complexity factors influence model performance, including the number of examples per class ($k_{trn}$), number of classes per episode (*n*-way), and number of unseen classes (*n*-unseen) were further analyzed. Additionally, the effects of image resolution, connectivity patterns, and computational requirements on each of the evaluated architectures were explored.

The adoption of the GFSL paradigm aligns more closely with the clinical presentation of pathologies in chest X-rays than the SFSC paradigm. This task formulation is particularly well-suited to specialized medical contexts and is useful for a variety of scenarios within the healthcare domain. In addition, the proposed multi-label episode generation algorithm enables the creation of complex classification tasks, further broadening its applicability in real-world medical settings. Interestingly, the results show that BatchBased is an effective classification method in few-shot scenarios, despite being based on STL and not specifically designed for few-shot learning. Furthermore, increasing the number of classes per episode (*n*-way) and the number of training examples per class ($k_{trn}$) improves the model performance by enhancing task robustness. With respect to the image resolution, using resolutions higher than those commonly applied in natural image tasks improves classification performance. This improvement can be attributed to the fine-grained nature of pathology classification, in which abnormal patterns are subtle and can be overlooked at lower resolutions. However, this performance improvement comes at the cost of higher computational demands and longer training and inference times. By contrast, the results show that efficient architectures can achieve a performance comparable to that of larger models while substantially reducing the computational overhead. This is particularly advantageous in resource-constrained environments such as remote areas or small hospitals, where these architectures strike a balance between performance, computational efficiency, and practical deployability.

In future work, four main research directions are envisioned. First, vision foundation models can be leveraged as a starting point for pathology classification. Pre-trained on large-scale datasets, these models can provide richer and more generalizable feature representations, thereby enhancing classification performance. Second, multi-modal classification models that integrate complementary information from radiology reports, such as radiologist notes and clinical records, can be developed. Incorporating this additional contextual information can enrich the diagnostic process and improve the overall performance. Third, the behavior of ProtoNet-ML can be analyzed under different distance and activation functions. Finally, conducting a comparative evaluation between the model predictions and expert radiologist assessments. This study can enable the clinical validation of the results and provide a more accurate and reliable measure of the model’s effectiveness in clinical settings.

## Data Availability

A general description of the MetaChest dataset is provided at https://bereml.github.io/metachest/, whereas the code needed to generate the dataset can be found at https://github.com/bereml/metachest. In addition, the code necessary to reproduce our main results is publicly available at https://github.com/bereml/meta-cxr.

## Abbreviations

CT: Computed tomography
GFSC: Generalized few-shot classification
GFSL: Generalized few-shot learning
HM: Harmonic mean
MAC: Multiply-accumulate operation
MTL: Meta-learning
ROC-AUC: Receiver operating characteristic-area under the curve
SFSC: Standard few-shot classification
SFSL: Standard few-shot learning
STL: Standard transfer learning
TL: Transfer learning
